# Editorial: Nutraceutical formulations and natural compounds for the management of chronic diseases

**DOI:** 10.3389/fmed.2026.1975614

**Published:** 2026-09-07

**Authors:** Vincenzo Piccolo, Anis Ben Hsouna, Maria Maisto

**Affiliations:** 1 Department of Pharmacy, University of Naples Federico II, Naples, Italy; 2 Department of Environmental Sciences and Nutrition, Higher Institute of Applied Sciences and Technology of Mahdia, University of Monastir, Monastir, Tunisia

**Keywords:** chronic diseases, clinical translation, healthy aging, natural bioactive compounds, nutraceuticals

## Introduction

The widespread of chronic diseases has improved the interest in nutraceuticals and natural bioactive compounds as complementary strategies for disease prevention and management. Food-derived bioactives can influence biological processes involved in inflammation, oxidative stress, immune regulation, metabolic homeostasis, gut microbiota function, and cellular signalling. Consequently, nutraceutical research has expanded from the identification of promising phytochemicals to a multidisciplinary field involving food chemistry, molecular biology, pharmacology, formulation science, epidemiology, and clinical medicine (1, 2). However, the clinical translation of nutraceuticals and natural compounds remains limited. Poor bioavailability, inadequate formulation strategies, limited standardization of botanical preparations, regulatory heterogeneity, and the relatively small number of high-quality clinical studies continue to restrict their application (3, 4). In addition, the multifactoriality of chronic diseases requires research approaches that combine robust mechanistic investigations with innovative epidemiological methodologies and well-designed clinical trials. Addressing these limitations requires better-standardized products, stronger evidence from clinical studies, and a closer connection between mechanistic and clinical research. In this context, the articles collected in this Research Topic address different aspects of these challenges, from the characterization of bioactive compounds and their mechanisms of action to formulation development, methodological advances, and clinical investigation.

Natural compounds, evidence generation, and clinical translation.

Over the past decade, nutraceutical research has expanded beyond the identification of biologically active compounds. Several contributions in this Research Topic highlight that natural compounds exert their biological effects through the modulation of multiple interconnected pathways, simultaneously influencing inflammation, oxidative stress, immune responses, metabolism, and other processes involved in the development and progression of chronic diseases (5). The review by Yu et al. provides an extensive overview of the pharmacological properties of flavonoids across a wide range of pathological conditions, including inflammatory signalling, apoptosis, angiogenesis, oxidative stress, and immune function. The review also highlights the need to preserve the structural integrity of bioactive compounds during extraction and processing to ensure product quality, reproducibility, and therapeutic efficacy. This multi-target activity may be particularly relevant to healthy aging, which is influenced by the interplay between chronic low-grade inflammation, oxidative stress, mitochondrial dysfunction, and metabolic alterations. In this regard, Numa et al. discuss the role of dietary polyphenols in promoting healthy aging through antioxidant, anti-inflammatory, epigenetic, and microbiota-mediated effects. The authors also discuss how advances in omics technologies, digital technologies, and food engineering are defining personalized nutritional strategies and the food industry is progressively moving toward precision nutrition tailored to the specific physiological characteristics of individual consumers. While Yu et al. and Numa et al. primarily focus on the biological activities of specific classes of natural compounds, Fatima et al. broaden the discussion by examining nutraceutical research from a translational perspective. Their review emphasizes that demonstrating biological activity alone is not sufficient to support clinical application and discusses the technological and regulatory challenges that remain to be addressed. Among these, bioavailability remains one of the most critical barriers, driving the development of innovative delivery systems designed to improve the stability, absorption, and biological performance of natural compounds, including nanoformulations, liposomes, hydrogels and bioenhancers (6). At the same time, the review highlights that advances in formulation technologies should be accompanied by standardized production processes, rigorous quality assessment, appropriate regulatory frameworks, and robust clinical validation. However, clinical translation requires evidence from well-designed experimental and clinical studies. In this regard, the studies published in this Research Topic reflect this transition, highlighting the complementary role of evidence synthesis, innovative methodological approaches, and clinical investigation in supporting the development of scientifically validated nutraceutical strategies. The relevance of evidence synthesis is illustrated by the systematic review and meta-analysis reported by Qin et al., which evaluates the effects of saffron supplementation on inflammatory and oxidative stress biomarkers in patients with type 2 diabetes mellitus. By quantitatively integrating the available clinical evidence, the authors provide a more balanced assessment of saffron's therapeutic potential. Although supplementation was associated with reduced circulating TNF-α levels, the current evidence remains inconclusive for several other biomarkers. Their work therefore not only provides clinically relevant information but also highlights the necessity of larger, well-designed randomized controlled trials able to overcome the limitations of the currently available literature. A different perspective on evidence generation is provided by Hou et al., who review the application of Mendelian randomization to investigate the causal relationships between insomnia and a broad spectrum of chronic diseases. By using genetic variants as instrumental variables, this approach reduces many of the limitations associated with conventional observational studies, allowing a more reliable assessment of causality. Although the review is not specifically focused on nutraceutical interventions, it highlights the growing importance of methodological approaches able to strengthen the quality of biomedical evidence. Due to the evolution of nutraceutical field, innovative strategies able to distinguish causal relationships from simple associations will be increasingly valuable for identifying preventive targets and supporting the development of more effective intervention strategies. The clinical study by Ganz et al. offers an example of how nutraceutical research is beginning to translate into patient-oriented investigations. Evaluating the effects of an innovative multi-fiber formulation in individuals with chronic gastrointestinal symptoms, the authors reported improvements in bowel regularity together with reductions in bloating and gas, providing preliminary evidence of its potential clinical benefit. Although further randomized controlled trials will be necessary to confirm these findings, the study reflects the need to move beyond mechanistic and preclinical observations toward interventions evaluated directly in human populations.

Although the studies included in this Research Topic differ in scope and methodology, they collectively reflect the direction in which nutraceutical research is evolving. Together, they highlight the importance of integrating mechanistic studies, technological innovation, evidence synthesis, and clinical investigation to build a stronger scientific foundation for nutraceutical interventions and support their translation into clinical practice.

## References

[1] Korber S Siedlok F Callagher L Elsahn Z. “Chapter 5 - Competitive advantage through multidisciplinary innovation in nutraceuticals: from concept optimisation to context transformation”. In: Santini C, Supino S, Bailetti L, editors. Case Studies on the Business of Nutraceuticals, Functional and Super Foods. Woodhead Publishing (2023). p. 85–104. 10.1016/B978-0-12-821408-4.00007-9

[2] Hajzer ZE Alibrahem W Kharrat Helu N Oláh C Prokisch J. Functional foods in clinical trials and future research directions. Foods (2025) 14:2675. 10.3390/foods14152675 40807611 PMC12346611

[3] Ashfaq R Rasul A Asghar S Kovács A Berkó S Budai-Szűcs M. Lipid nanoparticles: an effective tool to improve the bioavailability of nutraceuticals. Int J Mol Sci (2023) 24:15764. 10.3390/ijms242115764 37958750 PMC10648376

[4] Karthikeyan M Mehta A Kumar SS Mohan H Usha B. “Safety, dosage, and regulatory aspects in the use of phytochemicals”. In: Kuca K, Patocka J, Kumar V, Dhalaria R, editors. Medicinal Plants and Their Bioactives in Human Diseases. Cham: Springer Nature Switzerland (2025). p. 207–33 10.1007/978-3-032-01356-9_10

[5] Scotti L Scotti MT. Natural multi-targets: advances and applications. Curr Top Med Chem (2022) 22:1457–9. 10.2174/156802662218220727122830 36028941

[6] Yang B Dong Y Wang F Zhang Y. Nanoformulations to enhance the bioavailability and physiological functions of polyphenols. Molecules (2020) 25:4613. 10.3390/molecules25204613 33050462 PMC7587200

